# Developing biomarker assays to accelerate tuberculosis drug development: defining target product profiles

**DOI:** 10.1016/S2666-5247(24)00085-5

**Published:** 2024-05-09

**Authors:** Stephen H Gillespie, Andrew R DiNardo, Sophia B Georghiou, Wilber Sabiiti, Mikashmi Kohli, Ursula Panzner, Irina Kontsevaya, Norbert Hittel, Lieven J Stuyver, Jia Bin Tan, Reinout van Crevel, Christoph Lange, Thuong Nguyen Thuy Thuong, Jan Heyckendorf, Morten Ruhwald, Norbert Heinrich

**Affiliations:** **Division of Infection and Global Health, School of Medicine, University of St Andrews, St Andrews, UK** (Prof S H Gillespie DSc, W Sabiiti PhD); **Global TB Program, Texas Children’s Hospital and Baylor College of Medicine, Houston, TX, USA** (A R DiNardo PhD, C Lange MD); **Department of Internal Medicine and Radboud Center for Infectious Diseases, Radboud University Medical Center, GA Nijmegen, Netherlands** (A R DiNardo, R van Crevel PhD); **FIND, Campus Biotech, Geneva, Switzerland** (S B Georghiou PhD, M Kohli PhD, J B Tan MSc, M Ruhwald PhD); **Division of Infectious Diseases and Tropical Medicine, University Hospital, Ludwig-Maximilians-Universität München** (LMU), Munich, Germany (U Panzner MSc, N Heinrich PhD); **German Center for Infection Research (DZIF), Partner Site Munich, Munich, Germany** (U Panzner, N Heinrich); **Fraunhofer Institute for Translational Medicine and Pharmacology ITMP, Immunology, Infection and Pandemic Research, Munich, Germany** (N Heinrich); **Divisionof Clinical Infectious Diseases, Research Center Borstel, Borstel, Germany** (C Lange); **German Center for Infection Research (DZIF),Clinical Tuberculosis Unit, Borstel, Germany** (C Lange); **German Center for Infection Research (DZIF), Partner Site Hamburg-Lübeck-Borstel-Riems, Borstel, Germany** (I Kontsevaya PhD); **Department of Infectious Disease, Faculty of Medicine, Imperial College London, London, UK** (I Kontsevaya); **Janssen Global Public Health R&D, Janssen Pharmaceutica NV, Beerse, Belgium** (N Hittel PhD); **Otsuka Novel Products, Munich, Germany** (L J Stuyver PhD); **Centre for Tropical Medicine and Global Health, Nuffield Department of Medicine, University of Oxford, Oxford, UK** (R van Crevel); **Oxford University Clinical Research Unit, Ho Chi Minh City, Viet Nam** (T N T Thuong PhD); **Respiratory Medicine & International Health, University of Lübeck, Lübeck, Germany** (J Heyckendorf PhD, C Lange); **Clinic for Internal Medicine I, University Hospital Schleswig-Holstein (UKSH) Campus Kiel, Kiel, Germany** (J Heyckendorf)

## Abstract

Drug development for tuberculosis is hindered by the methodological limitations in the definitions of patient outcomes, particularly the slow organism growth and difficulty in obtaining suitable and representative samples throughout the treatment. We developed target product profiles for biomarker assays suitable for early-phase and late-phase clinical drug trials by consulting subject-matter experts on the desirable performance and operational characteristics of such assays for monitoring of tuberculosis treatment in drug trials. Minimal and optimal criteria were defined for scope, intended use, pricing, performance, and operational characteristics of the biomarkers. Early-stage trial assays should accurately quantify the number of viable bacilli, whereas late-stage trial assays should match the number, predict relapse-free cure, and replace culture conversion endpoints. The operational criteria reflect the infrastructure and resources available for drug trials. The effective tools should define the sterilising activity of the drug and lower the probability of treatment failure or relapse in people with tuberculosis. The target product profiles outlined in this Review should guide and de-risk the development of biomarker-based assays suitable for phase 2 and 3 clinical drug trials.

## Introduction

Tuberculosis drug development is hindered by the many limitations in the methods used to define outcomes in people with tuberculosis, especially the slow growth of *Mycobacterium tuberculosis* and the difficulty in obtaining suitable representative samples from patients throughout their treatment.^1,2^ In the case of pretomanid, this journey took almost 20 years.^3^

The drug development process (figure) includes dose finding^4^ and combination building,^5^ which often occurs in short 2-week phase 2a early bactericidal activity trials.^6–8^ Phase 2b studies extend the treatment period to 2 months, to assess the initial safety and efficacy of the regimens, with the most promising regimens then moving on to pivotal phase 3 studies.^9^ During this period, innovative approaches such as multi-arm and multistage designs can accelerate the drug development process by testing multiple drugs, doses, or combinations simultaneously.^10,11^ Developments in the methodology of drug trials have further expanded phase 2b studies to include end-of-treatment and longer-term outcome data (ie, relapse), with such trials being designated as phase 2c.^9^ Each phase 2 study design includes a measure of treatment response (such as time to first negative culture) or change in bacterial load as the primary study endpoint. This endpoint could be supplemented by linking the bacterial response directly to the pharmacokinetics of the drug in trial.^12,13^

Several promising new and potent drugs against tuberculosis are now available for evaluation, and the accelerated development of these agents requires improved methods that allow for rapid testing. Unfortunately, the evaluation process for these new agents is slow and demanding,^14^ including the determination of not only the optimal dose but also the most effective and safe combination of partner drugs over long study durations.

The length, arduousness, and resource-intensiveness of the process can partly be explained in terms of the need to monitor treatment response (ie, viability of *M tuberculosis*) as the trial endpoint for many patients with tuberculosis over long study durations. For this reason, drug trials require substantial investment in setting up and managing high-containment laboratories (biosafety level 3) and a strong cadre of scientists who can work in that environment. Furthermore, the growth-based reference standard is imperfect, further complicating the implementation of such techniques.^15^ Bacterial culture, especially *M tuberculosis* culture, is compromised by phenotypic lag, which is the failure or delay of growth on artificial media,^16–18^ and competition with rapidly growing organisms found in the sputum. Despite optimal decontamination procedures, the proportion of samples lost to contamination increases as the treatment progresses,^2^ which leads to frequent loss of the crucial final sample to contamination at the end of the treatment, effectively nullifying the contribution of the participant to the trial.^1^ The diagnostic methods available for treatment monitoring and their challenges are summarised in tables 1 and 2.

### Developing and evaluating more effective biomarkers

Biomarkers hold great promise in overcoming many of the challenges of culture-based endpoints (ie, time to result and contamination with other bacteria), and serve as early indicators of the efficiency of tuberculosis within the first 2 months of treatment and within long-term patient outcomes.^2,19^ Despite this potential of biomarkers to accelerate the evaluation of novel drugs and regimens against tuberculosis, a few been evaluated systematically for their ability to directly or indirectly correlate with the effect of early-phase treatment or with long-term outcomes in people with tuberculosis.^2,19^

To tap into this potential and accelerate drug development, UNITE4TB, a Europe-wide European Union-Innovative Medicines Initiative (EU-IMI)-funded research and industry consortium mandated to accelerate the development of novel drugs against tuberculosis,^20^ prioritised research on developing tools for monitoring tuberculosis treatment. The diversity of the potential biomarkers (which measure either the host response or the pathogen), sample types, and approaches for assay development and clinical assessment render comparisons of biomarker suitability and performance for assessing the effectiveness of drug treatment challenging. To solve this dilemma, optimal and minimal criteria should be defined to evaluate the design and performance of biomarker-based assays for monitoring tuberculosis treatment, especially in the context of trials for drugs against tuberculosis.

We, therefore, sought to define key specifications such as intended use, performance, operational characteristics, and pricing in a consensus-based target product profile (TPP), to guide the development of biomarkers in alignment with the specific needs of tuberculosis drug trialists. Of note, the assay requirements for monitoring treatment efficacy in clinical drug trials differ from those for monitoring treatment efficacy in clinical practice. This work is, therefore, complementary to the recently published WHO TPPs for programmatic management of tuberculosis in high-burden settings.^14,21^ In formulating the criteria for TPPs, we considered the possibility that the new developments might challenge current concepts regarding the design and performance of biomarker-based assays and therefore intentionally kept many of the criteria broad, to accommodate for future innovations while ensuring that the outlined characteristics would meet the needs of drug developers, clinical trialists, and reference laboratories involved in drug development and evaluation.

Although many studies have collected quantitative data by means of a range of techniques such as measurement of colony forming units,^7,8^ Mycobacteria Growth Indicator Tube (MGIT) time to positivity,^4,5^ Xpert,^22^ or molecular bacterial load assay,^2^ this Review is the first attempt to draft a TPP to quantify treatment response in clinical trial settings. Here, we consolidate the evidence that can allow the community of trialists and diagnostics developers in the field of tuberculosis to engage in debating the next steps. The TPP can enable assay developers to adjust their assays to meet the needs of tuberculosis drug developers, while enabling the drug development community to shorten the duration of clinical trials and recruit fewer people with tuberculosis to achieve statistically significant results, thus reducing the costs of advancing novel drugs and regimens rapidly.

### Search strategy and selection criteria

To focus our efforts on the drug development process, we brought together a team of individuals representing academic institutions, research institutes, small-sized and medium-sized enterprises, public organisations, and pharmaceutical companies within the biomarkers work package of the UNITE4TB consortium. As part of the development of TPP drafts, the trials consortium discussed the overarching purpose of the assays to monitor tuberculosis treatment, the aims to be met, and the necessary operational and performance characteristics of tools for tuberculosis drug development. The first task was to review the relevant literature and scope the landscape, which was achieved by means of literature searches in PubMed for articles published between January, 1990, and June, 2023, with the following search terms: “tuberculosis”, “clinical trials”, “monitoring”, “biomarkers”, and “treatment response”. No language filters were applied. We also reached out to academic colleagues and those working in commercial entities actively engaged in biomarker development and evaluation. In addition, crucial discussions were held with trialists, statisticians, and regulatory representatives, to arrive at the optimal performance parameters taking into consideration the current reference standard (ie, growth-based culture methods).

For each category, we outlined the desirable minimal and optimal criteria for acceptable tuberculosis treatment monitoring tools. In parallel with the development of quantitative bacterial biomarkers, the number of immune and inflammatory response assays under development has substantially increased, and some of them are starting to come to the market.^22,23^ The aim of these assays is to identify a measure of cure on the basis of normalisation of the immune and inflammatory status. The treatment response biomarkers that operate by this methodology and the relevant TPPs will be reviewed in a separate paper. We concluded, therefore, that there was a need to develop two TPPs, for tests that quantitatively assess viable tuberculosis bacteria, mainly for early-stage tuberculosis treatment response, and assess the late-stage tuberculosis treatment response that predicts the clinical long-term outcome of relapse-free cure in the context of trials for drugs against tuberculosis.

### Consultation and sense-checking

Draft 0 of the two TPP documents was distributed to 28 select individuals from the UNITE4TB consortium with experience in conducting tuberculosis drug trials or having backgrounds in tuberculosis biomarker research, or both, including individuals affiliated with ongoing tuberculosis efforts at Radboud University Medical Center (Radboudumc; Nijmegen, Netherlands), Ludwig-Maximilians-Universität München (LMU; Munich, Germany), Helmholtz Zentrum München Deutsches Forschungszentrum für Gesundheit und Umwelt (Neuherberg, Germany), KNCV Tuberculosis Foundation (KNCV; The Haag, Netherlands), Critical Path Institute Ltd. (C-PATH; Dublin, Ireland), The University of Porto (Porto, Portugal), The National University of Singapore (Queenstown, Singapore), GlaxoSmithKline Investigación y Desarrollo S L (GSK; Madrid, Spain), The Janssen Pharmaceutical Companies of Johnson & Johnson (Beerse, Belgium), Otsuka Novel Products (Munich, Germany), Oxford University Clinical Research Unit (OUCRU; Ho Chi Minh, Viet Nam), Research Center Borstel (Sülfeld, Germany), TASK Clinical Research Centre (Cape Town, South Africa), Instituto de Saude Publica da Universidade do Porto (ISPUP; Porto, Portugal), French National Research Institute for Sustainable Development (IRD; Marseille, France), Vita-Salute San Raffaele University (UniSR; Milan, Italy), and University College London (UCL; London, UK). The purpose of this initial internal review was to gather feedback on the outlined minimal and optimal performance criteria of biomarker-based assays for monitoring tuberculosis treatment in each TPP. All the feedback received was considered by the work package members, and the comments selected following several rounds of revision were incorporated into the final TPP documents. By the final revision, when a consensus was not reached between the work package members and internal reviewers on any performance criterion or metric, additional feedback and notes to explain any differences were added to the TPPs drafted.

Once all internal feedback was incorporated into the two TPPs, we selected an external panel of nine experts to provide another round of feedback on the draft documents through an online survey, with the goal of reaching a field consensus. Participants without declared or perceived conflicts of interest were selected for this exercise, while ensuring a broad representation of stakeholders with experience in tuberculosis biomarker research, including physicians and clinicians with experience in running treatment trials, public health practitioners with WHO and other policy experience, and experts from relevant settings with high disease burden. The final consensus was achieved through guided discussions with these external stakeholders on the available TPP drafts and the incorporation of survey results that defined needs with respect to the operational and performance characteristics.

### Characteristics of the TPPs

During preparation of the TPPs, the following points were addressed: scope or intended use, pricing, expected performance, and operational characteristics of biomarker-based assays for tuberculosis drug development. Clinical trials of novel tuberculosis agents are usually performed in a high-burden setting in a low-income or middle-income country, which has a substantial effect on the choice and application of the biomarker tests; thus, this consideration was included. Both optimal and minimal criteria were defined for each characteristic of the proposed tests, to prevent perfect from being the enemy of good (or practical).

The TPP outlines the minimal criteria defined for a listed characteristic, considered the lowest acceptable specifications that could be applied for that characteristic in a clinical trial. Similarly, the optimal criteria were defined as the ideal specifications to move the field forward. In developing these criteria, we intended that the optimal and minimal criteria would define the range of acceptable assay performance and operational characteristics. Although assay developers would be expected to design their assays to meet the optimal criteria listed for each specification, an assay would still be expected to hold value even without meeting these criteria under the condition that the assay presents sufficient advantages, especially when compared with the current reference standard. Thus, we underlined the importance of focused research to replace or reduce the dependence on culture-based endpoints, as these endpoints are compromised by sample loss due to contamination with faster growing organisms, which results in the loss of data points and wastage of the trialists’ and patients’ investments in the trial. Moreover, many people with tuberculosis are unable to produce a satisfactory sputum later in the treatment. Once a person with tuberculosis has culture-converted, the inability to produce a satisfactory sputum later in the treatment might not represent cure, and this limitation, together with the variability of defining culture positivity, can make inter-trial comparisons difficult.

Although sputum is the main sample used to define treatment outcome, the fact that some people, including women and most children, are unable to produce a satisfactory sample is less than ideal. Similarly, identification of a satisfactory sample for the evaluation of extrapulmonary tuberculosis, such as tuberculosis meningitis or osteomyelitis, is difficult. Reliance on sputum excludes those crucial populations from trials that have an urgent need for evidence of effective treatment regimens. Progress in this area is being achieved through the use of alternative specimen types for diagnosis, such as blood-based, stool-based, or urine-based tools.^24,25^

### Early-phase biomarkers

In the case of assays suitable for early-stage clinical trials for drugs against tuberculosis (table 3), the proposed criteria correlate with those accepted by regulators as phase 2b trial endpoints (table 1). In the context of phase 2 trials for the development of antimycobacterial compounds, the goal of the assay is to accurately quantify or reflect the number of viable bacilli in a patient sample as an indication of the efficacy of a drug or drug regimen in a trial. Although sputum samples would be an acceptable sample type, a non-invasive clinical specimen was seen as preferable, given the difficulty in obtaining sputum from many patients, especially those from clinically vulnerable populations (eg, people living with HIV and children). The setting, target users, and target population, along with most of the outlined operational characteristics, would be consistent with the clinical laboratories and laboratory conditions associated with drug trials.

For pricing considerations, the price of the current reference standard (ie, MGIT) was considered, although in the context of clinical trials, a notably higher price was recognised as acceptable under the condition that time would be saved in the conduct of the study (eg, lower sample size) or the non-responders to treatment would be identified at earlier timepoints. However, depending on the performance and operational characteristics, the potential market can be expanded to the settings of clinical trials as well. The expected performance metrics were kept broad to accommodate the diversity of the potential assays, while ensuring that the test accurately quantifies or reflects the number of viable bacilli in patient samples and their decline with effective treatment, with a dynamic range reflecting that found in the target sample. The assay would be expected to show results with high intra-laboratory and inter-laboratory agreement.

A concordance correlation coefficient against the reference standard was recommended to be determined for quantitative assays. Ultimately, assays would be expected to show advantages over the current culture-based reference standard in terms of the quantitative assessment of viable tuberculosis bacteria over the course of patient treatment, with focus on a quantitative output in correlation with treatment efficacy and a quicker time to result.

### Late-phase biomarkers

Developing a biomarker-based assay for use in phase 2c and pivotal phase 3 trials is most likely to be more challenging than developing one for use in the earlier stages of drug development, owing to the composite nature of the unfavourable outcome that serves as the study endpoint (table 4).^1,26,27^ Traditionally, a combined endpoint is used in evaluative studies, which includes a marker of successful treatment (ie, culture negativity at the end of the treatment) and the absence of recurrent infection with the initial infecting strain (defined as relapse). Within this definition, a degree of complexity exists, which should be addressed by distinguishing recurrence from relapse by sequencing the initial and recurrent strains obtained from the patient and applying the predefined criteria, which requires long-term storage of these isolates.^28–30^

To ensure the suitability of assays for late-stage clinical trials for drugs against tuberculosis (table 4), these assays were expected to accurately quantify or reflect the number of viable bacilli in a sample, to provide an indication of the efficacy of a drug or drug regimen in a trial (ie, absence of live organisms, as a definition of cure). Alternatively, repeated measures of viable count could be used to predict the outcome by means of methods that include mathematical modelling. Given the absence of methodologies that can predict the outcome, our criteria were intentionally kept broad to allow for technologies that are not yet known. Optimally, however, the assays should predict a relapse-free cure, replacing sputum culture conversion endpoints. As for early-stage assays, sputum samples would be an acceptable sample type, with non-invasive clinical specimens being more preferable, and the setting, target users, target population, and many of the outlined operational characteristics consistent with the settings associated with the drug trials.

### Price and cost

For pricing considerations, the price of the current reference standard (ie, MGIT) was again taken as a baseline. Quantifying and reflecting the number of viable bacilli were assumed to be cheaper methodologies to predict relapse-free cure. The expected performance metrics between biomarker-based assays enumerating bacilli and those predicting long-term outcomes would differ, with performance parameters of quantitative assays matching those of assays suitable for earlier-stage trials. Biomarker-based assays to identify the long-term cure in comparison with relapse in phase 2 or phase 3 trials would optimally show a good prediction of treatment outcome at earlier stages of treatment, as compared with culture in a per-trial arm analysis, with the minimal TPP criteria defined as the measurement at the end of the 12-month patient observation period being at least equivalent to culture and possibly having other advantages.^31^ Otherwise, performance parameters were again kept broad to accommodate the diversity of potential assays, with the expectation that the assays would show advantages over the current culture-based reference standard in terms of prediction of treatment efficacy and show a shorter time to result than the current culture methods. Innovative solutions using expensive tests such as imaging could be cost-effective in the context of a clinical trial, but their use would need to be justified by means of economic costing models and operational studies.

### Regulatory considerations

To replace current tuberculosis treatment regimens with improved, shorter, effective, all-oral regimens incorporating new drug compounds, the costs should be lowered and the clinical evaluation of new drugs and drug regimens accelerated. Although some progress has been made in this field following the introduction of new tuberculosis diagnostics, including Xpert MTB/RIF Ultra (Cepheid, Sunnyvale, CA, USA)^32^ and BACTEC MGIT (BD MGIT, Becton Dickinson, Franklin Lakes, NJ, USA),^15^ these technologies have not noticeably decreased the time or resources needed to conduct the drug trials, and the tuberculosis treatment monitoring methods currently in use have modestly improved the conduct of clinical trials, when compared with those of trials conducted 50 years ago.^33^ Of note, the requirements for monitoring treatment response in clinical trials are different from those in clinical practice; thus, the TPPs presented in this paper should complement those recently published by WHO.^14,21^

To accelerate drug development and offer improved therapeutic options to people with tuberculosis, there is a need to move beyond the accepted reference standard biomarker-based assay for tuberculosis detection and treatment monitoring—ie, sputum culture conversion. Culture has the advantage of being recognised as a US Food and Drug Administration-regulated technology with an in-vitro diagnostic use label (eg, bioMérieux BacT/ALERT 3D–BD MGIT–Thermo Scientific VersaTREK). Culture conversion can serve as a surrogate endpoint, a marker that itself does not provide a direct measurement of the clinical benefit but either predicts or is reasonably likely to predict the clinical benefit, in clinical trials.^15^ Thus, the increase in time to positivity is regularly used as a surrogate of the bacterial load.^26,34,35^ Similarly, a decline in the signal from DNA-based PCR methodologies such as Xpert MTB/RIF Ultra correlates with the bacterial load,^36^ although the relationship between the bacterial count and the signal measured by means of PCR and culture is known to diverge as the tuberculosis treatment progresses.^37,38^

The absence of an unequivocal definition of cure for people with tuberculosis is the biggest challenge in developing a suitable biomarker for monitoring treatment in tuberculosis trials. Although methods to quantify bacterial load in early-stage trials are straightforward and several mathematical methods are available to analyse these data,^13,39^ for pivotal phase 3 trials, bacteriological cure is defined differently, relying on culture negativity, while disregarding the fact that relapse can occur at any time after thecompletionoftherapy.^1,26,27^ To address this discrepancy, a post-treatment period has been defined, although any isolate should be sequenced to differentiate relapse from re-infection, and mixed infection with another more resistant strain can also preclude accurate result interpretation.^30,40^ Similarly, in case an unequivocal definition of the immunological signature of cure is not available for host-dependent biomarkers, relying upon a pattern of markers associated with cure might provide a more likely probability of a favourable outcome.^22,23^ Such an approach has not yet been recognised by regulatory authorities.

## Conclusions and future work

The TPPs that we have developed and presented for biomarker-based assays that can be used to monitor response to experimental therapy in drug trials are intended to stimulate discussions in the clinical trial community, provide a better picture of the tools necessary to improve trialling methodology, and ultimately accelerate regimen development and evaluation. Notably, suitable biomarker-based assays meeting the TPP criteria are anticipated to at least match the current standard of serial measurements of MGIT time to positivity, to monitor the response to therapy, considering that these methods are prone to contamination with oral bacteria that cause false positive results and prevent tuberculosis detection. Thus, a major goal of a new biomarker could be to obviate the need for sputum culture. Over the past 20 years, several technologies have been proposed for this purpose, including serial measurements of specific mRNA, 16S ribosomal RNA, or mycobacterial lipoarabinomannan.^2,27–29,41,42^ Some of these techniques are in the late stages of development and are undergoing regulatory trials for tuberculosis treatment monitoring.

These TPPs reflect the current perspectives of diagnosticians, clinical trialists, microbiologists, and others in setting aspirational criteria for monitoring the effect of tuberculosis treatment and accelerating clinical trials for drugs against tuberculosis. In addition to considering the valuable inputs from experts in this field, we strove to keep some performance criteria sufficiently broad to remain mindful of current and future inventions and developments that we might not be aware of currently. Ultimately, the two TPPs (suitable for early-stage and late-stage drug trials) were closely aligned with one another with regard to the target population (ie, drug trial participants with pulmonary tuberculosis), target users and setting (laboratory scientists in well equipped laboratories), pricing, repeatability and reproducibility, dynamic range, sample type, and operational performance characteristics as suitable for clinical laboratories participating in trials for drugs against tuberculosis. Notably, the TPPs differed in their ultimate goal, with the optimal criterion for the TPP for late-stage tuberculosis treatment response markers being an accurate prediction of relapse-free cure, whereas that for early-stage tuberculosis treatment response markers being accurate quantification of the number of viable bacilli in a sample obtained from the participant.

These TPPs represent consensus criteria that could be used to guide and de-risk the development of biomarker-based assays suitable for phase 2 and 3 clinical drug trials. Tools meeting the outlined minimal and optimal criteria would be expected to identify regimens with higher sterilising activity and lower probability of treatment failure or relapse in people with tuberculosis, thus revolutionizing the conduct of trials for drugs against tuberculosis. We believe that these TPP criteria will be useful to diagnostic developers and clinical trialists, stimulating innovation and further research leading to the launch of new and improved tools for the development of drugs against tuberculosis.

## Figures and Tables

**Figure:**
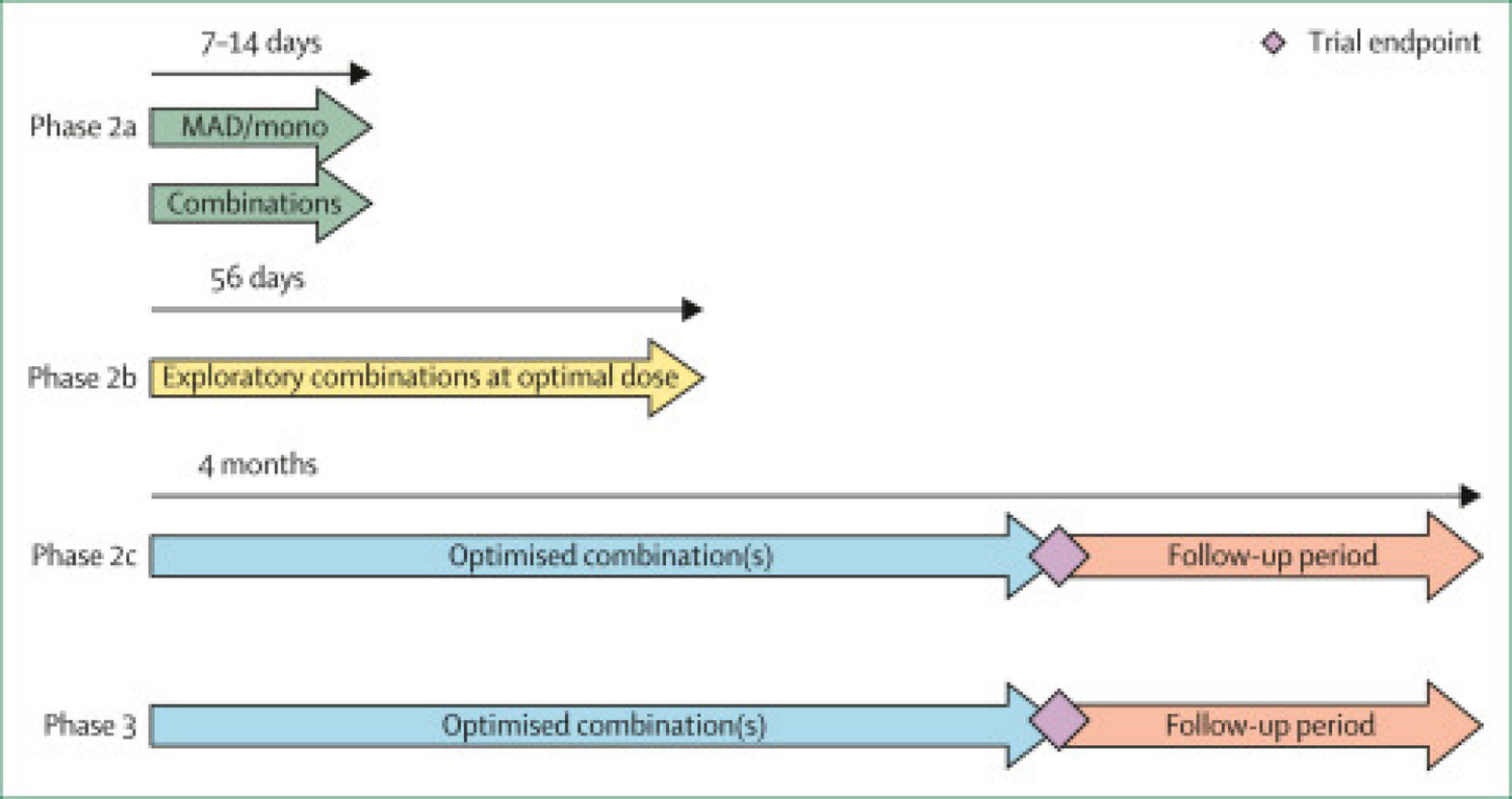
Tuberculosis drug development process An overview of the timelines and assay methodology in the tuberculosis drug development process.

**Table 1:** Summary of samples, methodologies, and endpoints used to measure early-stage outcomes in trials for drugs against tuberculosis

	Challenges

**Microbiological samples**	
Overnight sputum*	High contamination in ambient temperature; difficulty in obtaining specimen(s); biohazard risk.
**Microbiological methods**	
CFU on solid agar†	Semi-quantitative; prone to contamination; absolute count reduced by the effect of selective medium and decontamination methods; long time to result; high day-to-day variation; requirement of technical expertise to perform CFU counts; BSL3 laboratory facilities.
Becton Dickinson BACTEC MGIT‡	Semi-quantitative; prone to contamination; absolute count reduced by the effect of selective medium and decontamination methods; long time to result; correlation to CFU changes over the treatment, suggesting the need for assessment of different bacterial populations.
**Primary endpoint**	
Daily change TTP (day 0–14)	Compromised by sample loss: a contaminated sample results in the loss of a data point.
**Secondary endpoints**	
EBA (day 0–2)	Compromised by sample loss: a contaminated sample results in the loss of a data point.
EBA (day 2–14)	
EBA (day 0–7)	
EBA (day 7–14)	

BSL3=biosafety level 3. CFU=colony-forming unit. EBA=early bactericidal activity. MGIT=Mycobacteria Growth Indicator Tube. TTP=time to positivity.

* Spot samples are a recognised alternative.

† Lowenstein–Jensen medium was used as a diagnostic and antibiotic-containing agar-based media for quantitative counts.

‡ Manufactured product incorporating automated growth detection.

**Table 2:** Summary of the samples, methodologies, and endpoints applied to late-stage outcomes

		Challenges

Culture-based endpoints in phases 2b and 2c	Rate of change in MGIT TTP over the treatment (usually from day 0 to 56 or later).	Compromised by sample loss due to contamination and the consequent loss of data points; high day-to-day variation; no information on the effect of the treatment, after the culture converts to negative (goes below the limit of detection of MGIT).
	Time on treatment to sustained negative MGIT culture (sputum culture conversion, as defined by EMA^14^).	Compromised by sample loss: a contaminated sample results in the loss of a data point; inability of patients to produce sputum later in the treatment; does not reflect treatment efficacy after the culture converts to negative; several ways to define this parameter, making inter-trial comparisons difficult.
	Proportion of patients with negative cultures after 2 or more months of the treatment.	Compromised by sample loss: a contaminated sample results in the loss of a data point (entire patient); binary endpoint, leading to larger sample sizes.
Clinical endpoints: long-term cure *vs* unfavourable outcome: duration ranging from phase 2c to phase 3	Sustained cure *vs* relapse.	Outcomes occur late after randomisation and require long-term follow-up with patients, causing issues such as loss to follow-up and low clarity in dealing with intercurrent, mostly unrelated events such as deaths or premature terminations of study participation.
	Relapse (bacteriologically confirmed).	Relapse might be defined as the return of microbiologically confirmed tuberculosis with the same strain that caused the first episode of the disease, identified on the basis of appropriate typing methods (genome sequencing); distinguishing relapse from new infection might be difficult^15^ —EMA suggests that the case should then be counted as a relapse in the primary analysis of efficacy (re-infection would not be seen as an unfavourable outcome owing to unsuccessful treatment). However, this process could lead to an underestimation of the difference between the two treatment arms.
	Treatment failure (bacteriologically confirmed).	Treatment failure might be defined as deficient on-treatment sputum culture conversion at a pre-specified timepoint after the commencement of therapy or at the end of the treatment. The challenge is to distinguish persistent positivity (failure) from isolated single-positive cultures that often occur in patients responding well to treatment, but do not indicate failure of treatment; here, clinical observation on the resolution of signs and symptoms should be used.^15,16^ Separately, patients whose symptoms and possibly radiological evidence indicate a recurrence with or without microbiological confirmation might be judged as a failure of the clinical treatment.

EMA=European Medicines Agency. MGIT=Mycobacteria Growth Indicator Tube. TTP=time to positivity.

**Table 3:** Summary of the proposed criteria for a target product profile that can be used for assays that assess the number of viable tuberculosis bacteria in an early-stage tuberculosis clinical trial

	Optimal	Minimal	Explanation

Intended use	In-vitro (laboratory) research-use-only test for detecting viability markers of *Mycobacterium tuberculosis,* to aid in the assessment of early efficacy during phase 2 trials for the development of antimycobacterial compounds.		This definition is open to any potential microbiological solution by means of any method and would include but not be restricted to previously published methods for assessed viability.
Goal of test	A marker or combination of markers to accurately quantify or reflect the number of viable bacilli in a sample obtained from a patient with tuberculosis, to indicate the efficacy of a drug or drug regimen in a trial.		
Target population	All patients with active pulmonary tuberculosis evaluated in an early-phase trial.	Adults with drug-susceptible pulmonary tuberculosis.	
Target user of test	Laboratory scientists with the ability to perform low-complexity to moderate-complexity assays.	Laboratory scientists with the ability to perform high-complexity assays.	For the definitions of low-complexity and moderate-complexity assays, refer to the terminology defined by WHO for tuberculosis tests. An example of a low-complexity assay would be Cepheid Xpert MTB/RIF Ultra; that of a moderate-complexity assay would be Roche Cobas MTB-RIF/INH; and that of a high-complexity assay would be assays similar to or more complex than line probe assays, including sequencing-based technologies. As the assays will be used in the context of a drug trial, the target users are expected to have training and experience appropriate for work in clinical laboratories.
Setting (health system level)	Well equipped laboratory with good clinical practice and EQA standards. Future development might include low-complexity assays that can be performed in high-burden remote settings.		Assays are expected to be used in clinical laboratories, as appropriate for drug trials, although the potential market can be expanded to the settings of clinical trials depending on the performance and operational characteristics.
Pricing of individual test (reagent costs only; at scale; ex-works)	<US$20 per specimen.	<$1000 per specimen.	Price corresponds to the cost of testing per specimen. Tests that directly replace quantitation of viable bacilli would ideally be in the lower part of this range.
Capital costs of instrumentation	<$10 000	<$100 000	Tests that directly replace quantitation of viable bacilli would ideally be in the lower part of this range. However, innovative solutions using more expensive capital equipment could prove to be cost-effective in the context of clinical trials but would need to be justified by means of economic costing and operational studies.
Tests to enumerate bacilli or replace quantitative culture for phase 2a trials and eventually phase 2b trials	The marker or combination of markers should quantify or reflect the number of viable bacilli in a clinical sample.		Given the range of biomarkers for monitoring the treatment of tuberculosis, in addition to the variability in clinical samples and imperfect reference standards, no specific performance metric is given. Developers and manufacturers should have data (for a sufficient number of samples and timepoints) in relation to EBA or culture, or both, to confirm that the test quantifies or reflects the number of viable bacilli in patient samples and shows decline over time of effective treatment.
Dynamic range of detection	For tests of viable bacteria, the dynamic range should mirror that found in the target samples from patients with tuberculosis. For example, for a sputum test, the range should be between 10^1^ CFU/mL and 10^7^ CFU/mL.		A concordance correlation coefficient should be determined for quantitative assays to assess the performance against a reference standard (EBA or culture, or both), as appropriate for the assay.
Repeatability and reproducibility	The marker or combination of markers should generate highly similar results when a sample is tested on multiple occasions in the same laboratory or in different laboratories (inter-laboratory agreement).		The methods and results of reproducibility determination should be acceptable to regulators.
Sample type	Non-invasive clinical specimens.	Sputum or non-sputum samples that are not more complex to obtain than sputum, or both.	Given that sputum samples can be difficult to obtain for specific populations (eg, people living with HIV and children), in addition to patients with tuberculosis, the test would ideally be performed in easy-to-obtain non-sputum samples, including blood, stool, exhaled breath, and urine samples.
Sample stability	The analyte should be stable for at least 8 h upon storage at 25°C and for up to 48 h upon storage at 5°C and tolerate at least two freeze and thaw cycles.	The analyte should be stable for same-day analysis upon storage at 5°C.	
Sample preparation and assay processing (total steps)	Integrated sample preparation and detection in a closed system with minimal technical input.	Fewer number of steps required for sample preparation and detection.	
Time to result	<24 h, with automated result interpretation.	<7 days	<7 days would still be considered a significant improvement over growth-based methods for early-phase studies. The optimal characteristic considers the time from patient sampling to the final result.
Result output	Automated result and interpretation. The results screen should be integrated into the technology and the ability to save and print the results should also be included; the device should have a commonly used port (eg, USB or USB-c) and the solution should be integrated with the existing laboratory management information system.	Results might require interpretation by means of software but should be available in 1 day. The results screen should be integrated into the technology and the ability to save the results should be included; the device should have a commonly used interface port (eg, USB or USB-c).	These criteria were defined considering the fact that laboratories at clinical trial sites are expected to have back-up generators for crucial pieces of equipment.
Power requirements	Standard operating currents with a built-in UPS for utilisation in locations with variable power. In addition, battery or solar operation for locations with power supply disruptions.	Standard operating currents with a built-in UPS for utilisation in locations with variable power.	Although the test is anticipated to be used in a clinical laboratory with sufficient infrastructure, including UPS, a battery or solar-operated device would be ideal for operation in any setting.
Maintenance	Preventive maintenance at 1 year or >1000 samples; include maintenance alert. Mean time to failure of at least 18 months.	Availability of included supplies and maintenance teams to provide technical assistance as needed.	Some envisioned solutions might not require maintenance; the most important element will be the availability of supplies and maintenance teams to provide technical assistance as needed.
Calibration	Calibration should be done on site and the test should include all the necessary reagents and equipment.		Calibration should be simple to perform on site.
Operating temperature	Between +3°C and +40°C at 70–90% relative humidity.	Between +4°C and +30°C at 70–90% relative humidity.	
Spare supplies (not included in the kit)	None		
Internal quality control	Internal full-process positive and negative controls along with result standardisation across laboratories.		In addition to EQA.
Training and education needs	<1 day	3–5 days	Training times as appropriate for clinical laboratory scientists.

CFU=colony-forming unit. EBA=early bactericidal activity. EQA=external quality assessment. UPS=uninterrupted power supply. USB=universal serial bus.

**Table 4:** Summary of the proposed criteria for use of a target product profile in late-stage clinical trials, including phase 2b, phase 2c, and pivotal phase 3 clinical trials, on tuberculosis

	Optimal	Minimal	Explanations

**Goal of test**	A marker or combination of markers to accurately predict relapse-free cure, which would be observed for up to 12–18 months after randomisation, or as required by regulatory agencies*.	A marker or combination of markers to quantify the number of viable bacilli in a sample obtained from a patient with tuberculosis, to be used as a replacement for sputum culture-conversion endpoints or endpoints that describe the change in bacterial load†.	The marker or combination of markers should address the limitations of the current gold standard technology mentioned in table 2, and in case of the optimal characteristics, predict relapse-free cure at an earlier timepoint than the microbiological reference. If the measurement of cure is not earlier, there would be a need for other operational advantages over culture.
**Target population**	All patients with active pulmonary tuberculosis evaluated in phase 2b, 2c, and 3 trials.	Adults with active, drug- susceptible pulmonary tuberculosis evaluated in phase 2b, 2c, and 3 trials.	
**Target user of test**	Laboratory scientists with the ability to perform low-complexity to moderate-complexity assays.	Laboratory scientists with the ability to perform high-complexity assays.	For definitions of low-complexity and moderate-complexity assays, refer to the terminology defined by WHO for tuberculosis tests. An example of a low-complexity assay would be Cepheid Xpert MTB/RIF Ultra, that of a moderate-complexity assay would be Roche Cobas MTB-RIF/INH, and that of a high-complexity assay would be assays that are similar to or more complex than line probe assays, including sequencing-based technologies. As the assays will be used in the context of a drug trial, target users are expected to have training and experience, as appropriate for work in clinical laboratories.
**Setting (health system level)**	Well equipped laboratory with good clinical practice and EQA standards.		Assays are expected to be used in clinical laboratories, as appropriate for drug trialling. Future development might simplify the methodology and allow for fewer demanding requirements.
**Pricing of individual test (reagent costs only; at scale; ex-works)**	<US$20 per specimen	<$1000 per specimen	Price corresponds to the cost of testing per specimen. Tests that directly replace quantitation of viable bacilli would ideally be in the lower part of this range. Innovative solutions using more expensive tests could prove to be cost-effective in the context of clinical trials, by shortening the time to result of a trial, allowing for more effective design of adaptive trials, reducing the number of non-evaluable patients, or otherwise lowering the sample size or time required, so their use would need to be justified by economic costing models.
**Capital costs of instrumentation**	<$10 000	<$100 000	Tests that directly replace quantitation of viable bacilli would ideally be in the lower part of this range. Innovative solutions using more expensive capital equipment could prove to be cost-effective in the context of clinical trials but would need to be justified in terms of economic costing and operational studies.
**Enumeration of bacilli or replacement of quantitative culture**	The marker or combination of markers should quantify the number of viable bacilli or other bacterial signatures in a clinical sample‡.		Given the range of biomarkers for monitoring of tuberculosis treatment, in addition to the variability in clinical samples and imperfect reference standards, no specific performance metric is given. Developers and manufacturers should have data (for sufficient number of samples and timepoints) in relation to the culture, to confirm that the test quantifies the number of viable bacilli in samples obtained from patients with tuberculosis and shows decline over time of effective treatment.
**Dynamic range of detection**	For tests of viable bacteria, the dynamic range should mirror that found in the target samples obtained from patients with tuberculosis. For example, for a sputum test, the range should be between 10 and 10^7^ CFU/mL^−1^.		A within-specimen *R*^2^ should be determined for quantitative assays, to define the performance against the reference standard (quantitative culture), as appropriate for the assay.
**Repeatability and reproducibility**	The marker or combination of markers should generate highly similar results when a sample is tested on multiple occasions in the same laboratory or in different laboratories (inter-laboratory agreement).		
**Timing of measurement or prediction**	Measurement during treatment or at the end of the treatment. Ideally, good predictive capability should be substantially earlier than at the end of the treatment.	Measurement at the end of the 12-month observation period post-randomisation.	If measurement is only attainable or predictive of cure late during the patient treatment, there should be other advantages to the use of the measurement that add benefit over current growth-based methods.
**Surrogacy on trial level, to predict long-term outcome**	The marker should predict the long-term outcome that is substantially superior to culture in a per-trial arm analysis.	The marker should predict a long-term outcome that is atleast as good as culture in a per-trial arm analysis.	The reference standard for long-term cure *vs* failure or relapse needs to be defined in accordance with regulatory (EMA or FDA) guidance, requiring isolate sequencing and confirmation of relapse by matching the sequence with that of the initial isolate.
**Sample type**	Non-invasive clinical specimens (including blood).	Sputum or non-sputum samples that are not more complex to obtain than sputum samples (eg, urine or blood), or both.	Given that sputum samples can be difficult to obtain for specific populations (eg, people living with HIV and children) and patients with tuberculosis who are undergoing treatment, the test would ideally be performed in easy-to-obtain non-sputum samples, such as aerosol and urine.
**Sample stability**	Analyte should be stable for at least 8 h upon storage at 25°C and for up to 48 h upon storage at 5°C and tolerate at least two freeze and thaw cycles.	Analyte should be stable for same-day analysis upon storage at 5°C.	
**Sample preparation and assay processing (total steps)**	Integrated sample preparation and detection in a closed system with minimal technical input.	Fewer number of steps required for sample preparation and detection.	
**Time to result**	<24 h, with automated result interpretation.	<7 days.	<7 days would still be considered a substantial improvement over growth-based methods for early-phase studies. The optimal characteristic considers the time from patient sampling to result.
**Result output**	Automated result and interpretation. The results screen should be integrated into the technology and the ability to save and print results should be included; the device should have a commonly used port (eg, USB or USB-c) and the solution should be integrated with the existing laboratory management information system.	Results might require interpretation through software but should be available in 1 day. The results screen should be integrated into the technology and the ability to save results should be included; the device should have a commonly used interface port (eg, USB or USB-c).	These criteria were defined with the expectation that the laboratories at the sites of the clinical trials have back-up generators for crucial pieces of equipment. Interfacing and data transfer to laboratory information management systems for electronic data transfer into the trial database is desirable.
**Power requirements**	Standard operating currents with a built-in UPS for utilisation in locations with variable power. In addition, battery or solar operation for locations with power supply disruptions.	Standard operating currents with a built-in UPS for utilisation in locations with variable power.	Although the test is anticipated to be used in a centralised clinical laboratory with sufficient infrastructure, including uninterrupted power, a battery or solar-operated device would be ideal for operation in any setting.
**Maintenance**	Preventive maintenance at 1 year or >1000 samples; should include maintenance alert. A mean time to failure of at least 18 months.	Availability of spare supplies and maintenance teams to provide technical assistance, as needed.	Some envisioned solutions might not require maintenance; the most important element will be the availability of supplies and maintenance teams to provide technical assistance, as needed.
**Calibration**	Calibration should be done on site and the test should include all the necessary reagents and equipment.		Calibration should be simple to perform on site.
**Operating temperature**	Between +3°C and +40°C at 70–90% relative humidity.	Between +4°C and +30°C at 70–90% relative humidity.	
**Spare supplies (not included in the kit)**	None		
**Internal quality control**	Internal full-process positive and negative controls, along with results standardisation across laboratories.		In addition to EQA.
**Training and education needs**	<1 day	3–5 days	Training times, as appropriate for clinical laboratory technicians.

CFU=colony-forming unit. EMA=European Medicines Agency. EQA=external quality assessment. FDA=US Food and Drug Administration. UPS=uninterrupted power supply. USB=universal serial bus.

* With the availability of shorter and more potent regimens, this time period is likely to be shortened and this characteristic will need to be updated.

† Removing the need for culture would substantially simplify the trial practice.

‡ The definition of “other marker” is open to any biologically plausible methodology for which adequate data are available.
